# Spiral bacteria in the human stomach: the gastric helicobacters.

**DOI:** 10.3201/eid0103.950302

**Published:** 1995

**Authors:** A Dubois

**Affiliations:** Department of Medicine, Uniformed Services University of the Health Sciences, Bethesda, Maryland 20814-4799, USA. dubois@usuhsb.usuhs.mil

## Abstract

During the past decade, Helicobacter pylori has become recognized as one of the most common human pathogens, colonizing the gastric mucosa of almost all persons exposed to poor hygienic conditions from childhood. It also is often found, albeit with a lower frequency, in groups of high socioeconomic status. H. pylori causes chronic active gastritis and is a major factor in the pathogenesis of duodenal ulcers and, to a lesser extent, gastric ulcers. In addition, the presence of this bacterium is now recognized as a risk factor for gastric adenocarcinoma and lymphoma. Nevertheless, most infections appear without clinical consequences. In this second decade of intensive research, it is important to understand why H. pylori is sometimes a dangerous pathogen, and to determine how it can be eradicated in those at highest risk for severe disease.


### Spiral Bacteria in the Human Stomach: The Gastric Helicobacters

**Andre Dubois, M.D., Ph.D.**
Digestive Diseases Division, Department of Medicine,
Uniformed Services University of the Health Sciences, Bethesda, Maryland, USA

*During the past decade,* Helicobacter pylori *has become recognized as one of the most common human pathogens, colonizing the gastric mucosa of almost all persons exposed to poor hygienic conditions from childhood. It also is often found, albeit with a lower frequency, in groups of high socioeconomic status.* H. pylori *causes chronic active gastritis and is a major factor in the pathogenesis of duodenal ulcers and, to a lesser extent, gastric ulcers. In addition, the presence of this bacterium is now recognized as a risk factor for gastric adenocarcinoma and lymphoma. Nevertheless, most infections appear without clinical consequences. In this second decade of intensive research, it is important to understand why* H. pylori *is sometimes a dangerous pathogen, and to determine how it can be eradicated in those at highest risk for severe disease.*

At the end of the 19th century, several types of spirochetes and spirilla were observed for the first time in the stomach of animals (1,2). Beginning at the turn of the 20th century, similar spiral bacteria were found in gastrectomy specimens from patients with gastric cancer and peptic ulcer disease (3,4). In addition, gastroenterologists and surgeons noted—but could not explain—the almost universal presence of antral gastritis in patients with duodenal ulcers and the frequent presence of atrophic gastritis in patients with gastric ulcer and cancer. Nevertheless, the possibility that peptic ulcer disease or gastric cancer might be caused by an infectious agent was generally discounted. The observation made in 1975 that gram-negative bacteria were present in 80% of patients with gastric ulcer (5) was largely ignored by the scientific community which, at the time, was busily developing potent antiulcer agents (6). Skepticism remained the overwhelming reaction to the 1983 reports describing the frequent association between antral gastritis and the presence of *Campylobacter*-like bacteria (7), as well as of their culture and isolation from patients with gastritis (8). A similar reaction followed the subsequent demonstration that these *Campylobacter*-like bacteria were present in almost all patients with gastric and duodenal ulcers, and were generally associated with antral gastritis (9). In the past decade, however, a number of studies have confirmed and extended these early observations. A consensus regarding the major role of this bacterium, now named *Helicobacter pylori*, in causing gastroduodenal ulceration was formally presented in 1994 (10). Furthermore, in June 1994, the International Agency for Research on Cancer Working Group stated , "*H. pylori* plays a causal role in the chain of events leading to cancer," referring to adenocarcinoma and lymphoma of the stomach as well as to the more benign mucosal-associated lymphoid tissues (MALT) (11-13).

An important consequence of the considerable interest generated by these clinical observations is that extensive bacteriologic and molecular studies have been performed on this bacterium and similar organisms. 16S rRNA gene sequence analysis has revealed important differences between *H. pylori* and the closely related *Campylobacter, Flexispira,* and *Wolinella* genuses. These differences have necessitated the creation of the genus *Helicobacter*, which, to date, includes eight gastric, three intestinal, and two hepatic species (14). Each of these *Helicobacter* species colonizes different, or a spectrum of, mammalian species.

This review summarizes our current knowledge of the two *Helicobacter* species that have been observed in the human stomach and reported on extensively in the literature: *H. pylori*, the type strain, and *H. heilmannii*, also known as *Gastrospirillum hominis* (15,16).

Address for correspondence: Andre Dubois, Department of Medicine, Uniformed Services University, 4301 Jones Bridge Road, Bethesda, MD 20814-4799, USA; fax: 301-295-3676 or -3557; e-mail dubois@usuhsb.usuhs.mil.

### Characteristics of Gastric Helicobacters Observed in Humans

*H. pylori*, a gram-negative bacterium with a curved, spiral, or gull-wing shape, is 2.5 to 3.5 µm long and 0.5 to 1.0 µm in diameter and has a periodicity of 1 to 2 µm. It has smooth surfaces, and one to six polar-sheathed flagellae emerge from one of its rounded ends. Since it is morphologically similar to *C. jejuni*, it was initially named "pyloric *Campy-*

*lobacter*" and subsequently *C. pyloridis* and *C. pylori* before finally being named *H. pylori*. This organism colonizes only the non-acid-secreting mucosa of the stomach and is not found where parietal cells are numerous. Thus, it may be observed in the gastric antrum and the cardia, but also in the corpus, when atrophic gastritis is present, and attached to the gastric epithelial cells found in the duodenum, when gastric metaplasia is present.

*G. hominis* (*H. heilmannii*) is tightly spiraled, and is 3.5 to 7.5 µm in length and 0.9 µm in diameter; it has a periodicity of 0.8 to 1 µm and up to 12 flagellae at each pole. 16S rRNA indicates that this organism belongs to the genus *Helicobacter*, and is more closely related to a *Helicobacter* sp. isolated from the stomach of cats (*H. felis*) than to *H. pylori* (17). The name *H. heilmannii* was proposed in honor of the late German pathologist Heilmann. However, the subsequent examination of the rRNA of different clinical isolates indicates that there is enough heterogeneity among isolates tentatively identified as *H. heilmannii* that it is premature to propose an official name (17) . This bacterium colonizes only the parietal cell area of the gastric mucosa and may be found within parietal cells (18,19).

### Diagnosis

*H. pylori* infection may be diagnosed by harvesting gastric biopsy specimens during endoscopy, by culturing and isolating the bacterium under microaerobic conditions (90% $N_2$, 5% $O_2$, and 5% $CO_2$), and by characterizing the enzymes (urease, catalase, and oxidase) it produces. Visualization of the bacterium by light microscopy on slides stained with hematoxylin and eosin, Gram, Giemsa, Genta, or Warthin-Starry stain is also of great benefit since it allows the concurrent diagnosis of the extent of the antral chronic-active gastritis that *H. pylori* causes. However, because *H. pylori* colonization is focal, negative biopsy results do not exclude the possibility of infection in areas not sampled. Infection also may be diagnosed by determining plasma and salivary immunoglobulin (Ig) G or IgA levels with enzyme-linked immunosorbent assays (20,21). This latter technique is noninvasive, specific, and sensitive and is believed to reflect the mucosal and systemic immunity induced by *H. pylori* infection.

Two other tests, which rely on the production of urease, also can be used to identify *H. pylori*. One is the CLO (for *Campylobacter*-like organisms) test, which is performed by placing a mucosal biopsy specimen in medium containing urea and a pH-sensitive dye that changes color in the presence of $OH^-$ ions. The second test is the noninvasive $^{14}C$ or $^{13}C$ breath test following the oral administration of $^{14}C$- or $^{13}C$-urea. Neither of these tests is specific for *H. pylori* since *G. hominis*, which generates urease, also gives a positive reaction. Until specific methods based on the polymerase chain reaction (PCR) amplification of 16S rRNA (17) become widely available, the diagnosis of *G. hominis* infection must rely on histologic morphologic characteristics; histologic identification must be confirmed by transmission electron microscopy since other spiral organisms, e.g., *Flexispira rappini*, also may be present in the stomach of humans (22).

### Epidemiology

The seroepidemiology of *H. pylori* has been extensively studied in the United States and in other countries (23). The high frequency of seropositivity (up to 100% in some age groups in Albania) and acquisition of the infection during infancy are characteristic of disadvantaged socioeconomic groups living in crowded or poor hygienic conditions and appears to be independent of gender and ethnic origin. In adults of higher socioeconomic groups, the rate of seroconversion is estimated at 0.5% per year, although the frequency of seropositivity increases with age and may be as high as 40%. A longitudinal study has indicated that the high frequency of seropositivity in older adults might be due to a higher rate of *H. pylori* infection in Western countries in the years between the two world wars than during recent years (cohort effect) (24). Alternatively, the increase in frequency of infection in older adults might be due to years of low but cumulative risk for infection. Although the route of transmission for this infection is not known, the contamination of drinking water may play a role in certain developing countries (25). In the United States and in other regions, direct contact and/or consumption of food or water contaminated by saliva (26), gastric contents, or feces (27) may be major factors. The recent observation that *H. pylori* can be isolated from cats (28) suggests that transmission from pets to humans (or humans to pets) is also possible.

The epidemiology and route of transmission of *G. hominis* are largely unknown. The frequency of this infection appears to range from less than 1% of the population in industrialized countries (29) to 3% to 8% in developing countries (30). Although the detection of spirilla in the stomach of cats and dogs suggests possible transmission from pets, marked morphologic differences exist between these spirilla and the organism found in the stomach of humans.

### Pathogenicity

*H. pylori* is considered a pathogen because its presence is always associated with chronic active gastritis, and eradication of the bacterium is always followed by resolution of gastritis. In addition, nearly all patients with duodenal ulcer disease have *H. pylori* gastritis, and ulcer relapse is exceptional after *H. pylori* eradication. Thus, the presence of *H. pylori* seems necessary for the production of duodenal ulcers, with the exception of ulcers attributed to the use of nonsteroidal antiinflammatory agents or to the Zollinger-Ellison syndrome (10). The association with gastric ulcers is not as strong, although *H. pylori* infection is present in 80% of patients with gastric ulcers who do not consume nonsteroidal antiinflammatory agents (10). However, most *H. pylori*-infected persons do not report any clinical symptoms. This may be because these persons are colonized by less virulent strains or because other host or bacterial cofactors are required for overt disease.

In addition, three prospective cohort studies have demonstrated that *H. pylori*-infected persons have an increased risk of developing intestinal-type, but not undifferentiated, gastric adenocarcinoma (10). In fact, the association of *H. pylori* with either gastric ulcer or gastric cancer may be underestimated in these studies: the atrophic gastritis that follows long-term infection makes the gastric niche less hospitable for the bacterium, which may either eliminate *H. pylori* or make it difficult to detect. Nevertheless, atrophic gastritis per se is believed to be a precancerous lesion that leads to carcinogenesis without the presence of *H. pylori*.

The pathogenicity of *G. hominis* is unclear. The organism has been associated with upper gastrointestinal complaints, and its carriage is generally accompanied by gastritis, although the inflammation and gastric atrophy are less than noted with *H. pylori* (31,32). In addition, *G. hominis* was observed in gastric cancer patients (3) as well as in patients with only minimal gastritis (29). In this relatively small number of cases, the frequent concurrent infection with *H. Pylori* makes interpreting the respective pathogenic role of either bacterium difficult. It is probable that G. *hominis* will turn out to be at least somewhat pathogenic, as it makes urease and products of urease action that have been implicated in inflammation.

### Colonization and Virulence Factors

*H. pylori* multiplies with great efficiency in the hostile environment within the stomach but survives poorly in the gastric lumen; it is mainly found where the pH ranges between 4 and 7, i.e., under the mucous layer and in close proximity, or even attached, to gastric superficial epithelial cells. The virulence and the ecologic niche of *G. hominis* are unknown, although its presence within parietal cells of patients with gastrointestinal complaints (18,19) suggests that it is even more resistant to acid than *H. pylori*.

The production of urease was the first putative colonization or virulence factor studied. The production of this enzyme is shared by the two organisms, and it may explain their extraordinary ability to survive in an environment previously considered sterile because of the presence of proteolytic enzymes, as well as the low pH of gastric contents. Because the ecologic niches of these bacteria are rich in urea, urease generates $OH^-$ ions that neutralize gastric acid. Although the neutralization of gastric acid benefits the two bacteria, the production of hydroxide ions also is toxic to gastric epithelial cells in vivo, as indicated by in vitro experiments (33).

Two other important virulence factors shared by *H. pylori* and *G. hominis* are their spiral shape and the motility of their flagellae, which render them resistant to peristaltic flushing of the gastric contents and enable them to persist in the mucous layer. Because *G. hominis* appears to infect fewer persons than *H. pylori*, a more important role might be attributable to characteristics that are unique to *H. pylori*; these include the production of other enzymes (catalase, oxidase, protease, and phospholipase), as well as the synthesis of specific adhesin proteins that enable them to adhere to mucous and epithelial cells, both in vivo and in vitro (34-36).

The putative virulence factor of *H. pylori* that has commanded the most attention during the past few years has been its vacuolating cytotoxin (*vacA* gene product). Intragastric administration of the toxin to mice causes some (but not all) of the tissue damage seen in *H. pylori*-infected persons (37). In addition, cytotoxin production is highly correlated with the production of a high molecular weight (120 to 128 kilodaltons) major protein antigen that is called cytotoxin-associated protein (*cagA*) and is not the toxin itself (38).

### Diversity of *H. pylori*

*H. pylori* isolates may differ with respect to each of the virulence factors described above; this diversity is likely to contribute to variation in colonization or disease. For example, urease-negative strains have been isolated, and the vacuolating cytoxin is produced by only a subset of *H. pylori* strains (*vacA*+ or tox+ strains) (39-41). This observation is probably clinically relevant because most or all strains from duodenal ulcer patients, and many strains from gastric cancer patients, produce cytotoxin, whereas only a fraction of strains from patients with gastritis alone produce the cytotoxin (42,43). This phenotypic diversity is mirrored in great diversity on the DNA level. Thus, only cytotoxin-producing strains contain the gene for this cytotoxin-associated protein (*cagA*) (38,42), although genetic tests have shown that *cagA* protein is not needed for toxin production (44). Strains that do not produce the 128-kDa *cagA* protein generally lack the entire *cagA* gene and additional neighboring genes. Although the function of the *cagA* region is unknown, its presence or absence is easily scored by hybridization or PCR and thus serves as an easy marker for probable cytotoxin production and possible virulence of *H. pylori* strains. Additional virulence factors are likely to be present. For example, another recently discovered region constitutes at least 21 kilobases of the *H. pylori* genome in hybridization experiments, and its presence is highly correlated with the presence of *cagA*: 39 of 40 strains lacking *cagA* also lacked this region, and 50 of 52 strains containing *cagA* contained this region. This newly discovered region is being called *cagII*, and the effort to sequence it is nearly complete (D. E. Berg, pers. comm.). Preliminary searches have identified several open reading frames with strong homologies to virulence functions from other microbes (45).

In addition to these extensively studied genes, genetic diversity of various *H. pylori* strains can be demonstrated by the use of two sensitive, efficient, and reliable PCR-based methods (46,47). This approach is particularly useful because it allows tracing of strains in epidemiologic studies.

### Infection and Immune Response

One of the most puzzling aspects of gastric infection with *H. pylori* is its persistence despite intense local and systemic immune responses. These immune responses are extremely complex and vary among infected humans. The systemic response is characterized by a marked increase in plasma IgG, which remains present for months after the infection has been cured. The local response includes the production of IgA, which binds to the surface antigens of *H. pylori* in vitro and coats the bacterium in vivo. In addition, infection is consistently associated with an intense inflammatory response and the infiltration of cells into the gastric mucosa. Although polymorphonuclear cells are often present, most cells in such infiltrates are mononuclear cells. Both B and T cells are present, and recent studies have indicated that the natural killer activity of peripheral blood lymphocytes can be increased by *H. pylori*, possibly by its stimulating the production of interferon and other cytokines (48). Thus, the long-term carriage of the infection may be related to the ability of the bacterium to influence the T-cell response. Fragmentary evidence also suggests that this infection can be abortive and cure spontaneously without the use of antibiotics (A. Dubois and D. E. Berg, unpublished).

On the other hand, the mucosal response may promote colonization, as indicated by the observation that patients with acquired immunodeficiency syndrome (AIDS) tend to have a lower rate of infection than aged-matched subjects who are negative for human immunodeficiency virus (49,50). The latter study (50) also demonstrated that AIDS patients had a different pattern of gastritis, characterized by greater mononuclear cell responses, fewer lymphoid follicles, and a greater prevalence of intestinal metaplasia. The immune response may also prevent the invasiveness of *H. pylori*, as suggested by the anecdotal but puzzling observation of invasive *H. pylori* infection in a patient with AIDS (51).

### Treatment

Although *H. pylori* is sensitive to many antimicrobial drugs in vitro, it is difficult to eradicate from the stomach. This may be ascribed to antibiotic breakdown by gastric acid, clearance by gastric emptying, and the difficult-to-penetrate mucous layer in which the bacterium resides. Resistance of *H. pylori* to specific antibiotics, especially metronidazole, is also frequent. Therefore, it is generally accepted that a combination of at least two, and possibly three, antimicrobial agents should be given for a minimum of 1 week. The regimen found to be most effective is the administration of amoxicillin (or tetracycline) plus metronidazole and bismuth subsalicylate 2 to 4 times a day for 2 to 3 weeks (52). The use of one antibiotic associated with an antisecretory agent, such as a histamine $H_2$ receptor antagonist, has given disappointing results. In contrast, the combination of a proton pump inhibitor ($H^+$-$K^+$ ATPase antagonist) with amoxicillin or acid-stable macrolides (clarythromycin or roxithromycin) appears more promising; a number of studies are being conducted to determine the optimal dose, duration, concomitant therapy, and cost-effectiveness of these compounds (53,54). Recently, it was shown that at least a 7-day course of any of these regimens is required to obtain a high (90%) cure rate, but that continuing treatment for more than 10 days does not significantly improve its efficacy. Finally, topical therapy for 1 h was recently tried with excellent results, albeit in only one center at this time (55). This treatment involves a 2-day administration of a mucolytic agent to dissolve the mucous layer and of a proton pump inhibitor. On the third day, a balloon is introduced into the second portion of the duodenum under fluoroscopic control, and a solution of pronase, amoxicillin, metronidazole, and bismuth subsalicylate is injected into the stomach, where it is left for 1 h. The presence of the duodenal balloon appears to prevent emptying of the antibiotics and the mucolytic agent, thus ensuring maximum efficacy of the therapy.

### Future Research

The past 12 years have seen extensive progress in research on *H. pylori* as a cause of chronic active gastritis, duodenal ulcer disease, and gastric cancer. This has been largely due to an unusual collaboration among gastroenterologists, pathologists, molecular geneticists, bacteriologists, and immunologists. However, our understanding of how *H. pylori* colonizes and causes diseases is far from complete, and it will benefit from studies performed in animal models that can be experimentally infected with *H. pylori* (56-59). In addition, no easily administered treatment leading to eradication of this bacterium in all patients is yet available, although a better knowledge of its physiology may lead to the development of such a "silver bullet." Studies in animals that are not naturally infected with *H. pylori* suggest possibilities for vaccines (56,57), and ongoing trials in nonhuman primates are exploring the possibility of immunizing hosts that can be naturally infected with this organism. Although the elimination of peptic ulcer disease and of certain forms of gastric cancer will require extensive and coordinated efforts from public health authorities, this goal now appears to be within the reach of the scientific and medical community.

### Acknowledgments

The author thanks Drs. P. Baker and D.E. Berg for their helpful comments and suggestions during the preparation of this review.

*Dr. Dubois is professor of medicine and surgery (research), assistant director, Digestive Diseases Division, and chief, Laboratory of Gastrointestinal and Liver Studies, F. Edward Hébert School of Medicine of the Uniformed Services University of the Health Sciences, Bethesda, Maryland. He studies the physiology and pathophysiology of gastric secretion and gastric emptying as well as the role of gastric infection with* H. pylori *in gastroduodenal diseases.*

### References

1. Rappin J. Contribution à l'étude de bactéries de la bouche à l'état normal. 1881. Quoted by Breed RS, Murray EGD, Hitchens AP, Bergey's manual of determinative bacteriology, 6th ed. Baltimore: Williams & Wilkins, 1948;217.
2. Bizzozero G. Sulle ghiandole tubulari del tube gastroenterico e sui rapporti del loro coll'epitelio de rivestimento della mucosa. Atti R Accad Sci Torino 1892;28:233-51.
3. Krienitz W. Ueber das Auftreten von Spirochäten verschiedener Form im Mageninhalt bei Carcinoma ventriculi. Dtsch Med Wochenschr 1906:28:872-89.
4. Freedburg AS, Barron LE. The presence of spirochetes in human gastric mucosa. Am J Dig Dis 1940;7:443-5.
5. Steer HW, Colin-Jones DG. Mucosal changes in gastric ulceration and their response to carbenoxolone sodium. Gut 1975;16:590-7.
6. Black JW, Duncan WAM, Durant CJ, Ganelin CR, Parson EM. Definition and antagonism of histamine $H_2$ receptors. Nature 1972;236:384-90.
7. Warren JR. Unidentified curved bacilli on gastric epithelium in active chronic gastritis. Lancet 1983;i:1273.
8. Marshall BJ. Unidentified curved bacilli on gastric epithelium in active chronic gastritis. Lancet 1983;i:1273-5.
9. Marshall BJ, Warren JR. Unidentified curved bacilli in the stomach of patients with gastritis and peptic ulceration. Lancet 1984;i:1311-5.
10. NIH Consensus Conference. *Helicobacter pylori* in peptic ulcer disease. JAMA 1994;272:65-9.
11. Parsonnet J, Friedman GD, Vandersteen DP, et al. *Helicobacter pylori* infection and the risk of gastric carcinoma. N Engl J Med 1991;325:1127-31.
12. Nomura A, Stemmerman GN, Chyou PH, Kato I, Perez-Perez GI, Blaser MJ. *Helicobacter pylori* infection and gastric carcinoma in a population of Japanese Americans in Hawaii. N Engl J Med 1991;325: 1132-6.
13. Isaacson PG, Spencer J. Is gastric lymphoma an infectious disease? Hum Pathol 1993;24:569-70.
14. Fox JG, Yan LL, Dewhirst FE, et al. *Helicobacter bilis sp.* nov., a novel *Helicobacter* species isolated from bile, livers, and intestines of aged, inbred mice. J Clin Microbiol 1995;33:445-54.
15. Heilmann KL, Borchard F. Gastritis due to spiral shaped bacteria other than *Helicobacter pylori:* clinical, histological and ultrastructural findings. Gut 1991;32:137-40.
16. McNulty CAM, Dent JC, Curry A, et al. New spiral bacterium in gastric mucosa. J Clin Pathol 1989;42:585-91.
17. Solnick JV, O'Rourke J, Lee A, Tompkins LS. Molecular analysis of urease genes from a newly identified uncultured species of *Helicobacter.* J Infect Dis 1993;168:379-83.
18. Rollason TP, Stone J, Rhodes JM. Spiral organisms in endoscopic biopsies of the human stomach. J Clin Pathol 1984;37:23-6.
19. Dye KR, Marshall BJ, Frierson HF, Guerrant RL, McCallum RW. Ultrastructure of another spiral organism associated with human gastritis. Dig Dis Sci 1989;34:1787-91.
20. Perez-Perez GI, Dworkin BM, Chodos JE, Blaser MJ. *Campylobacter pylori* antibodies in humans. Ann Intern Med 1988;109:11-7.
21. Drumm B, Perez-Perez GI, Blaser MJ, Sherman PM. Intrafamilial clustering of *Helicobacter pylori* infection. N Engl J Med 1990;322:359-63.
22. Archer JR, Romero S, Ritchie AE, et al. Characterization of an unclassified microaerophilic bacterium associated with gastroenteritis. J Clin Microbiol 1988;26:101-5.
23. Taylor DN, Blaser MJ. The epidemiology of *Helicobacter pylori* infection. Epidemiol Rev 1991;13:42-59.
24. Cullen DJE, Collins BJ, Christiansen BJ, et al. When is *Helicobacter pylori* infection acquired? Gut 1993;34:1681-2.

25. Klein PD, Graham DY, Gaillour A, Opekun AR, Smith EO. Gastrointestinal Physiology Working Group. Water source as risk factor for *Helicobacter pylori* infection in Peruvian children. Lancet 1991:337:1503-6.
26. Ferguson DA, Li C, Patel NR, Mayberry WR, Chi DS, Thomas E. Isolation of *Helicobacter pylori* from saliva. J Clin Microbiol 1993;31:2802-4.
27. Thomas JE, Gibson CR, Darboe MK, Dale A, Weaver LT. Isolation of *H. pylori* from human faeces. Lancet 1992;340:1194-5.
28. Handt LK, Fox JO, Dewhirst FE, et al. *Helicobacter pylori* isolated from the domestic cat: public health implications. Infect Immun 1994;62:2367-74.
29. Mazzuchelli L, Wilder-Smith CH, Ruchti C, Meyer-Wyss B, Merki HS. *Gastrospirillum hominis* in asymptomatic, healthy individuals. Dig Dis Sci 1993;38:2087-9.
30. Chen Z, Wang B, Xu H, et al. Spiral shaped bacteria in the human gastric biopsy. Hua-Hsi I Ko Ta Hsueh Hsueh Pao 1993;24:392-4.
31. Logan RPH, Karim QN, Polson RJ, Walker MM, Baron JH. *Gastrospirillum hominis* infection of the stomach. Lancet 1989;ii:672.
32. Morris A, Ali MR, Thomsen L, Hollis B. Tightly spiral shaped bacteria in the human stomach: another cause of active chronic gastritis? Gut 1990;31:134-8.
33. Smoot DT, Mobley HLT, Chippendaele GR, Lewison JF, Resau JH. *Helicobacter pylori* urease activity is toxic to human gastric epithelial cells. Infect Immun 1991;59:1992-4.
34. Boren T, Falk P, Roth KA, Larson G, Normark S. Attachment of *Helicobacter pylori* to human gastric epithelium mediated by blood group antigens. Science 1993;262:1892-5.
35. Fauchere J, Blaser MJ. Adherence of *Helicobacter pylori* cells and their surface components to HeLa cell membranes. Microb Pathol 1990;9:427-39.
36. Hemalatha SG, Drumm B, Sherman PJ. Adherence of *Helicobacter pylori* to human gastric epithelial cells in vitro. Med Microbiol Immunol 1991;35:197-202.
37. Telford JL, Ghiara P, Dell'Orco M, et al. Gene structure of the *Helicobacter pylori* cytotoxin and evidence of its key role in gastric disease. J Exp Med 1994;179:1653-8.
38. Tummuru MK, Cover TL, Blaser MJ. Cloning and expression of a high-molecular-mass major antigen of *Helicobacter pylori:* evidence of linkage to cytotoxin production. Infect Immun 1993;61:1799-809.
39. Figura N, Gugliemetti P, Rossolini, et al. Cytotoxin production by *Campylobacter pylori* strains isolated from patients with peptic ulcers and from patients with chronic gastritis only. J Clin Microbiol 1989;27:225-6.
40. Cover TL, Dooley CP, Blaser MJ. Characterization of and human serologic response to proteins in *Helicobacter pylori* broth culture supernatants with vacuolizing cytotoxin activity. Infect Immun 1990;58:603-10.
41. Cover TL, Blaser MJ. Purification and characterization of the vacuolating toxin from *Helicobacter pylori*. J Biol Chem 1992;267:10570-5.
42. Covacci A, Censini S, Bugnoli M, et al. Molecular characterization of the 128-kda immunodominant antigen of *Helicobacter pylori* associated with cytotoxicity and duodenal ulcer. Proc Natl Acad Sci USA 1993;90:5791-5.
43. Cover TL, Glupczynski Y, Lage AP, et al. Serologic detection of infection with *cagA*$^{+}$ *Helicobacter pylori* strains. J Clin Microbiol 1995;33:1496-500.
44. Tummuru MK, Cover T, Blaser M. Mutation of the cytotoxin-associated *cagA* gene does not affect the vacuolating cytotoxin activity of *Helicobacter pylori*. Infect Immun 1993;62:2609-13.
45. Akopyants NS, Kersulyte D, Berg DE. *cagII*, a new multigenic locus only present in the most virulent *Helicobacter pylori* strains. Abstracts of the 95th General Meeting of the American Society for Microbiology. Washington, DC, 1995:181, Abstract B-90.
46. Akopyanz N, Bukanov NO, Westblom TU, Kresovich S, Berg DE. DNA diversity among clinical isolates of *Helicobacter pylori* detected by PCR-based rapid fingerprinting. Nucleic Acid Res 1992;20:5137-42.
47. Akopyanz N, Bukanov NO, Westblom TU, Berg DE. PCR-based RFLP analysis of DNA sequence diversity in the gastric pathogen *Helicobacter pylori*. Nucleic Acids Res 1992;20:6221-5.
48. Tarkkanen J, Kosunen TU, Saksela E. Contact of lymphocytes with *Helicobacter pylori* augments natural killer cell activity and induces production of interferon. Infect Immun 1993;61:3012-6.
49. Edwards PD, Carrick J, Turner J, Lee A, Mitchell H, Cooper DA. *Helicobacter pylori*-associated gastritis is rare in AIDS: antibiotic effect or a consequence of immunodeficiency? Am J Gastroenterol 1991;86: 1761-4.
50. Steephen A, Raijman I, Schwarz P, et al. The spectrum of gastritis in Zambian patients with the acquired immunodeficiency syndrome. Gastroenterology 1995;108:A921.
51. Meiselman MS, Miller-Catchpole R, Christ M, Randall *E. Campylobacter pylori* gastritis in the acquired immunodeficiency syndrome. Gastroenterology 1988;95:209-12.
52. Chiba N, Rao BV, Rademaker JW, Hunt RH. Meta-analysis of the efficacy of antibiotic therapy in eradicating *Helicobacter pylori*. Am J Gastroenterol 1992;87:1716-27.
53. Logan RPH, Gummett PA, Schaufelberger HD, et al. Eradication of *Helicobacter pylori* with clarythromycin and omeprazole. Gut 1994;35:323-6.
54. Graham DY, Opekun AR, Klein PD. Clarythromycin for the eradication of *H. pylori*. J Clin Gastroenterol 1993;16:292-4.
55. Kimura K, Ido K, Saifuku K, et al. A 1-h topical therapy for the treatment of *Helicobacter pylori* infection. Am J Gastroenterol 1995;90:60-3.
56. Michetti P, Corthésy-Theulaz I, Davin C, et al. Immunization of BALB/c mice against *Helicobacter felis* infection with *Helicobacter pylori* urease. Gastroenterology 1994;107:1002-11.
57. Marchetti M, Arico B, Burroni D, Figura N, Rappuoli R, Ghiara P. Development of a mouse model of *Helicobacter pylori* infection that mimics human disease. Science 1995;267:1655-8.

58. Krakowka S, Morgan DR, Kraft WG, Leunk RD. Establishment of gastric *Campylobacter pylori* infection in the neonatal gnotobiotic piglet. Infect Immun 1987;55:2789-96.

59. Dubois A, Fiala N, Heman-Ackah LM, et al. Natural gastric infection with *Helicobacter pylori* in monkeys. A model for human infection with spiral bacteria. Gastroenterology 1994;106:1405-17.
